# Lipoprotein(a): the neglected risk factor in cardiovascular health

**DOI:** 10.3389/fcvm.2025.1710557

**Published:** 2026-01-09

**Authors:** Allister Irvine, Joanne Watt, Mary Jo Kurth, Laura Mooney, Jonathan Clark-McKellar, Tracey Keteepe-Arachi, John V. Lamont, Le Roy Dowey, Peter Fitzgerald, Mark W. Ruddock

**Affiliations:** 1 Randox Laboratories Ltd., Crumlin, United Kingdom; 2 Andarta Health, Fitzroy Square, London, United Kingdom; 3 John Radcliffe Hospital, Oxford, United Kingdom; 4 School of Biomedical Sciences, Ulster University, Coleraine, United Kingdom; 5 Randox Health GB, London, United Kingdom

**Keywords:** atherosclerosis, cardiovascular disease, Lipoprotein(a), Lp(a), risk assessment, screening, stroke, QRISK3

## Abstract

Lipoprotein(a) [Lp(a)] is a well recognised contributor in the development of cardiovascular disease. Unlike other lipoproteins, Lp(a) levels are primarily genetically determined, and in most individuals remain largely stable throughout life. Elevated Lp(a) is common in the general population, and various international guidelines now recommend at least one lifetime measurement of Lp(a) and its inclusion into an individual's cardiovascular risk assessment. Despite this, Lp(a) is still rarely measured, even in patients with known cardiovascular risk factors. Critically, the therapeutic landscape for Lp(a)-lowering medications is rapidly evolving with multiple drugs showing considerable promise in late-stage clinical trials. The strength and consistency of the evidence now cement Lp(a) as an essential biomarker of cardiovascular health. Failure to incorporate measurement of Lp(a) into clinical practice will continue to underestimate an individual's risk of CVD. Now is the time for Lp(a) to move from a neglected biomarker to a widely known and measured essential component of cardiovascular risk assessment.

## Introduction

Cardiovascular disease (CVD) is the leading cause of death worldwide, with over 19.4 million deaths reported in 2021 (1). It is estimated that by adapting to lifestyle changes, 75% of cardiovascular mortality can be reduced (2). However, for some individuals, a residual risk of CVD remains despite a reduction in traditional risk factors (3). Lipoprotein(a) [Lp(a)] has been established as an independent and causal risk factor for the development of CVD (4). Various guidelines including those issued by the European Atherosclerosis Society (EAS) and the National Lipid Association (NLA) recommend the measurement of Lp(a) in adults to identify individuals at high cardiovascular risk (5, 6). Despite this well-known relationship, Lp(a) is not routinely measured as part of cardiovascular risk assessments. In this perspective article, we argue that the omission of widespread Lp(a) testing represents a critical gap in contemporary cardiovascular risk assessments. We suggest that cardiovascular risk assessment must evolve from a narrow focus on modifiable lifestyle factors to a more comprehensive model that integrates the genetically determined risk, Lp(a). This article highlights historical barriers that have hindered widespread adoption of Lp(a) testing and outlines strategies to overcome them. Furthermore, this article discusses the rapidly evolving landscape of Lp(a)-lowering therapies, which may provide targeted benefit to individuals with elevated Lp(a) and residual cardiovascular risk. Considering the high proportion of the population who are estimated to have high Lp(a), and the potential approval of novel therapies, it is critical that Lp(a) is urgently incorporated into standard cardiovascular risk assessment.

## Lp(a) and its impact on cardiovascular health

Understanding of Lp(a) and its effects on cardiovascular health is currently limited among the general public. Lp(a) is a variant of low-density lipoprotein (LDL) cholesterol, which is synthesised in the liver (7). In contrast to LDL-cholesterol, which is strongly influenced by diet and pharmacotherapy, Lp(a) levels remain relatively stable throughout a person's life (7). Non-genetic factors can also influence blood levels of Lp(a), including comorbidities such as chronic kidney disease, thyroid dysfunction, acute inflammation and medications (5). Unlike other lipoproteins, lifestyle changes are typically ineffective at lowering Lp(a) levels.

Lp(a) contributes to CVD through four main mechanisms: atherogenesis, thrombosis, calcification, and vascular inflammation (8). Lp(a) is estimated to be 5–6 times more atherogenic than LDL-cholesterol (9). Lp(a) is the predominant carrier of oxidised phospholipids in blood; oxidised phospholipids activate the innate immune system causing inflammation and calcification (8). Lp(a) may also contribute to thrombosis through antifibrinolytic interaction with platelets due to structural similarities with plasminogen (9). Numerous large-scale studies have established that Lp(a) is a causal factor in the development of atherosclerotic cardiovascular disease (ASCVD) and aortic valve stenosis (4). Data from the Copenhagen General Population Study reported that elevated Lp(a) levels are associated with increased risk for the development of aortic valve stenosis, myocardial infarction, heart failure and ischaemic stroke (5, 6).

## Challenges with routine screening for Lp(a) in clinical practice

Despite research demonstrating the importance of measuring Lp(a) in cardiovascular risk assessments, Lp(a) is not routinely measured. For example, in a recent study of six medical centres in the University of California health system, Lp(a) testing was only undertaken for 0.3% of patients, and in <4% of patients with a personal history of cardiovascular disease (10). Similarly, in a German study of 2018 health records from 9 million patients, only 0.34% of these individuals received an Lp(a) test (11). The lack of routine Lp(a) measurements may be due, in part to the multiple challenges associated with the measurement and reporting of Lp(a).

The first major challenge in the adoption of Lp(a) is linked to the measurement variability in the size of the Lp(a) isoforms. The molecular weight of Lp(a) isoforms can vary from 275 to 800 kDa due to variability in the size of the apolipoprotein(a) domain (12). As Lp(a) immunoassays typically use polyclonal antibodies which bind to the apolipoprotein(a) domain, detection of Lp(a) varies widely depending on the antibodies used, with Lp(a) typically underestimated in individuals with small isoforms and overestimated in those with larger isoforms (13). These differences in Lp(a) detection contributed to conflicting results in early Lp(a) population studies where the relationship between Lp(a) levels and cardiovascular risk was unclear because of the use of isoform sensitive assays (12). Historically, these differences in measurement reduced confidence in Lp(a) as a consistent marker of CVD. Modern Lp(a) immunoassays rely on the use of multiple calibrators which span a large range of Lp(a) concentrations and apolipoprotein(a) isoforms to determine Lp(a) levels more accurately and mitigate isoform size differences (7). Although no Lp(a) assay is entirely isoform insensitive, currently assays based on Denka Seiken reagents, which employ the use of five calibrators, that contain a range of apolipoprotein(a) isoforms, are regarded as the most isoform insensitive (12). The Northwest Research Lipid Laboratory in the University of Washington provides an Lp(a) certification process which compares the performance of Lp(a) immunoassays to a monoclonal antibody-based ELISA (12). A remaining issue concerns the internationally accepted calibrator material (WHO/IFCC SRM-2B) for Lp(a) which is almost depleted (14). A new mass spectrometry-based reference has been developed (15) with new serum reference materials estimated to be provided by the International Federation of Clinical Chemistry and Laboratory Medicine in 2025 (16). It is critical that once new standards are available, that Lp(a) assays are updated and aligned to the new material to ensure consistency of Lp(a) measurements.

Differences in the measurement units reported by different immunoassays are the second challenge in the adoption of Lp(a). Historically, Lp(a) assays were reported in mass units, which incorrectly assume that the mass of Lp(a) proteins are consistent (7). To account for this, consensus guidelines now recommend reporting of Lp(a) in molar units (17). While conversion factors between molar and mass units exist, these conversions are approximate estimates and may be inaccurate depending on an individual's Lp(a) isoform size and are generally not recommended (5). These inconsistencies in Lp(a) reporting have impacted the confidence in use of Lp(a) in clinical practice with results given in different units contributing to confusion with risk interpretation but adherence to the consensus guidelines will mitigate this issue.

In efforts to standardise the measurement of Lipoprotein(a), HEART UK, a UK-based cholesterol charity, issued a consensus statement in 2019 with recommendations on the measurement of Lp(a) in laboratories (18). These recommendations suggest: (i) Lp(a) should be measured using a method with appropriate antibodies where the effect of isoform size is minimised and calibrators are certified to the WHO/IFCC reference material, (ii) Lp(a) concentrations are reported in nmol/L units, (iii) conversion between mass and molar units is inaccurate and should be discouraged and, (iv) the use of assays using Denka-based reagents with WHO/IFCC reference material reported in nmol/L (18). However, in a 2021 survey of UK clinical laboratories, only 5% of laboratories had fully implemented the HEART UK recommendations with most laboratories unsure of the Lp(a) methods they were using (13). It is imperative that laboratories readily adopt the HEART UK recommendations to ensure that widespread Lp(a) testing is consistent and doesn't contribute to any further measurement confusion.

The third challenge in the adoption of Lp(a) is the lack of a universal consensus on what ‘cut-off’ level should be used to assign an individual as having high Lp(a) levels (9). Cardiovascular risk increases linearly with rising Lp(a) concentrations, but the absolute risk attributable to Lp(a) depends heavily on the presence of other cardiovascular risk factors, which makes determination of precise ‘cut-off’ levels challenging (19). Despite this, various groups have suggested different thresholds to determine increased risk of CVD for a given Lp(a) level. The EAS defined three Lp(a) risk categories based on Lp(a) level: rule-in risk [Lp(a) > 125 nmol/L or >50 mg/dL], grey zone [Lp(a) 75–125 nmol/L or 30–50 mg/dL] and rule out risk [Lp(a) < 75 nmol/L or <30 mg/dL] (5). Conversely, HEART UK defines four cardiovascular risk categories based on Lp(a) levels: minor risk (32–90 nmol/L; 18–40 mg/dL), moderate risk (90–200 nmol/L; 40–90 mg/dL), high risk (200–400 nmol/L; 90–180 mg/dL) and very high risk (400 nmol/L; 180 mg/dL) (17). In recent years, there have been some efforts to standardise cut-off thresholds. For example, in 2019 guidelines from the NLA, an Lp(a) level ≥100 nmol/L (≥50 mg/dL) cut-off was recommended to indicate high risk (20). In 2024, the NLA changed their recommendations to align with the EAS risk cut-off of >125 nmol/L (>50 mg/dL) (6). Differences in the clinical interpretation of Lp(a) risk thresholds continue to hinder the integration of Lp(a) into clinical practice guidelines. The incorporation of Lp(a) consensus risk thresholds into guidelines issued by clinical organisations such as the National Institute for Health and Care Excellence (NICE) would go a long way to consolidating clinical approaches to Lp(a) risk assignment.

Historically it has been challenging to measure Lp(a) for the reasons presented above, however, improvements in immunoassays, standards and clinical guidelines have mitigated these challenges to allow for confident routine measurement of Lp(a) as part of cardiovascular risk assessments. Importantly, measurement of Lp(a) using the most readily available assay (whether reported in molar or mass units) is preferable than no measurement of Lp(a) (21).

## Population burden of Lp(a) and its risk implications

Global modelling studies estimate that more than 1.8 billion people have an elevated Lp(a) level (22). Lp(a) concentrations vary across ethnic groups due to genetic differences. Black and South Asian individuals have higher median Lp(a) levels compared with White and Chinese individuals, based on data from the UK Biobank (19). Despite these population differences, the relative risk associated with elevated Lp(a) (>150 nmol/L) appears consistent across groups (19).

**Figure 1:**
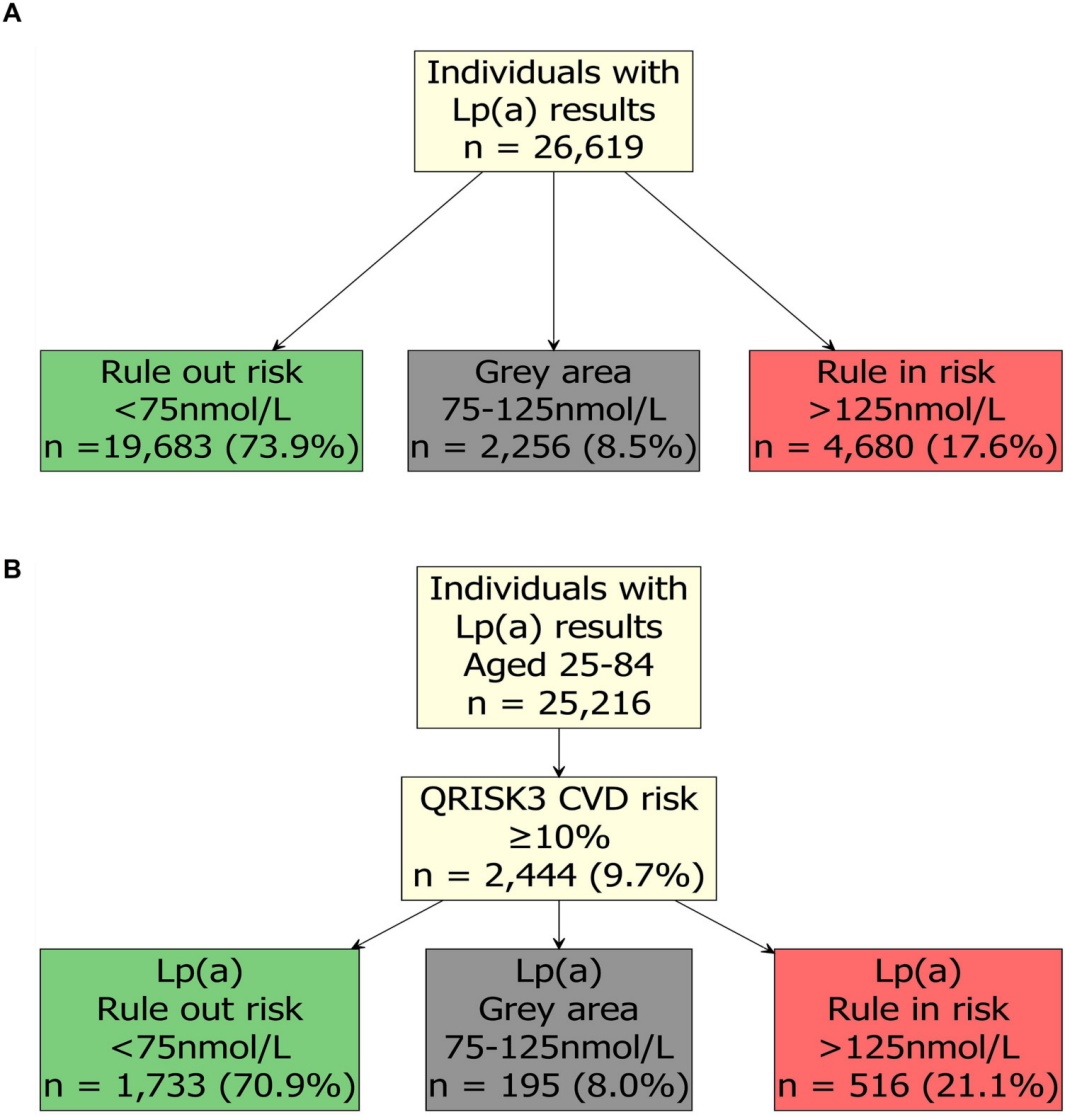
Lipoprotein(a) [Lp(a)] results from health-aware individuals. **(A)** Lp(a) results and risk level in health-aware individuals according to European Atherosclerosis Society (EAS) guidelines. **(B)** QRISK3 score and Lp(a) results in health-aware individuals according to NICE and EAS guidelines. Results were from individuals that attended a Randox Health Clinic for a health check within the UK between July 2023 and June 2025. Serum Lp(a) levels were determined by Randox Clinical Laboratory Services (RCLS; ISO17025 accredited) using a Lipoprotein(a) assay (LP3403, Randox Laboratories Ltd.) on an RX Imola analyser (Randox Laboratories Ltd.) and Lp(a) categories were assigned ‘rule out’ risk (green), ‘grey area’ (grey) and ‘rule in’ risk (red) based on the European Atherosclerosis Society guidelines (5). Consent was provided by the individuals for their data to be used for research purposes, and the analysis was reviewed and approved by Ulster University, School of Biomedical Sciences Ethics Filter Committee (Project Number: FCBMS-25-104-A). 10-year QRISK3 (2017) score was estimated using the QRISK3 package (https://cran.r-project.org/package=QRISK3).

Cardiovascular risk rises linearly with increasing Lp(a) concentrations, but the absolute risk depends strongly on the coexistence of other risk factors. As a result, current CVD risk assessment tools that do not incorporate Lp(a), such as QRISK3, may substantially underestimate true risk. We examined anonymised retrospective data from health-aware individuals who attended a Randox Health Clinic in the UK for general health screening checks. In this UK-based cohort, 17.6% (4,680/26,619) of individuals with Lp(a) results were identified as ‘rule in’, (increased risk of CVD), based on EAS guidelines (Figure 1A). A further 8.5% (2,256/26,619) had Lp(a) results in the ‘grey area’ (75–125 nmol/L), and 73.9% (19,683/26,619) were identified as ‘rule out’ (not at increased risk) (Figure 1A). Considering how common elevated Lp(a) is, it is important that at-risk individuals are made aware of their increased risk of CVD [based on Lp(a)], and the importance of the management of controllable CVD risk factors, such as elevated blood pressure, LDL-cholesterol, and glucose (5). Moreover, it may also be beneficial for individuals in the grey area to be retested, at a future date, for Lp(a); a recent study found that 53% of individuals in the grey area transitioned to different risk categories following a repeat Lp(a) measurement (23).

To simulate how these individuals would be assigned cardiovascular risk following UK NICE guidelines (24), we estimated their 10-year QRISK3 (2017) score using the QRISK3 package (https://cran.r-project.org/package=QRISK3) in R (25). As NICE only recommends use of QRISK3 for people aged 25–84, we excluded *n* = 1,403 individuals outside of this age range. In this cohort, 9.7% (2,444/25,216) had an estimated QRISK3 score of 10%, or greater (Figure 1B). For patients with a QRISK3 score of 10% or more, the standard approach to treatment in the UK is statin therapy alongside lifestyle modification (26). Alarmingly, in individuals who had a ≥10% QRISK3 score, 21.1% (516/2,444) also had a ‘rule in’ risk level of Lp(a) > 125 nmol/L (Figure 1B); these individuals may require more intensive lipid-lowering management and monitoring. Individuals in the ‘grey area’ [8.0% (195/2,444)] may require an additional test to define their Lp(a) risk.

## Inclusion of Lp(a) in cardiovascular risk assessments

Given its strong association with cardiovascular events, and the limitations of current risk models, there is a compelling case for including Lp(a) in CVD risk calculators such as QRISK3. The Lipoprotein(a) taskforce has called for standardisation of Lp(a) screening and measurement, inclusion of Lp(a) into CVD risk calculators, and inclusion of Lp(a) within clinical guidelines (27). Recently, at an Lp(a) Global Summit, the Brussels International Declaration on Lp(a) Testing and Management was published calling for integration of Lp(a) into Global Cardiovascular health plans, establishment of Lp(a) testing policy, and a commitment to ensure systematic Lp(a) testing is offered to all individuals, at least once (28).

Various studies have demonstrated that inclusion of Lp(a) levels into existing CVD risk calculators such as SCORE and PREVENT would improve estimation of cardiovascular risk (29–33). Inclusion of Lp(a) into pre-existing risk calculators would assist clinicians in identifying individuals at high risk who might otherwise be missed. Furthermore, early identification would allow for more aggressive management of modifiable risk factors, potentially reducing the risk of heart attacks and strokes.

Various online tools such as the Lp(a) clinical guidance calculator (https://www.lpaclinicalguidance.com/) have been developed to assess risk of heart attack and stroke by age 80. In a recent case study of a patient who had an abnormal lipid profile, and a family history of CVD, the patient's risk of heart attack or stroke was calculated as 17%. However, with the inclusion of Lp(a) in the risk calculation, the patient's updated risk of heart attack or stroke more than doubled, to 40% (34). Additionally, the Lp(a) clinical guidance calculator estimates how risk can be decreased by lowering blood pressure and LDL-cholesterol levels. As Lp(a) measurement becomes more integrated into clinical practice, visualisation tools will become important in helping clinicians and individuals to understand the risk and how lifestyle, or medication interventions can help to mitigate risk.

## Lp(a) treatment and novel therapies

A further complication in the clinical management of elevated Lp(a) levels is the lack of approved medication options to lower Lp(a) levels. Lipoprotein apheresis can significantly reduce Lp(a) levels by >60% (35), but is typically reserved for patients who have high cholesterol levels which are unresponsive to medication, or those with familial hypercholesterolemia (36). Additionally, lipid apheresis is limited to specialist lipid clinics, is expensive and requires repeat apheresis appointments (8, 35).

The most cost-effective approach to managing risk attributable to elevated Lp(a) involves comprehensive risk reduction through the optimisation of all other modifiable cardiovascular risk factors (5). This includes aggressive control of non-HDL cholesterol, blood pressure, blood glucose, and lifestyle factors such as smoking cessation, physical activity, and dietary improvements. Since Lp(a) levels are largely unaffected by lifestyle changes or conventional lipid-lowering therapies like statins, clinicians must focus on holistic cardiovascular risk management (37).

Reductions in Lp(a) can be achieved using proprotein convertase subtilisin kexin type 9 (PCSK9) inhibitors, such as alirocumbab and evolocumab. In trials evaluating evolocumab usage for LDL cholesterol reduction, *post hoc* analyses showed reductions in Lp(a) levels by 15.5%–31.3% (9). Similarly, alirocumab administration reduced Lp(a) levels by ∼30% on average (9). However, PCSK9 inhibitors were not developed to specifically target Lp(a) and have not been approved for lowering Lp(a) levels (38).

Although there is currently an absence of approved medications specifically targeting Lp(a), the therapeutic landscape is rapidly evolving. Several novel pharmacological agents which target Lp(a) synthesis are in various stages of clinical development, with many showing promise in significantly lowering Lp(a) levels (Supplementary Table S1). Three Lp(a)-lowering drugs which are currently undergoing phase III clinical trials are Pelacarsen, Olpasiran and Lepodisiran (38). All three therapies target liver synthesis of the apolipoprotein(a) mRNA, either as antisense oligonucleotide therapy (Pelacarsen) (39) or a small interfering RNA (Olpasiran and Lepodisiran). Each of the therapies have demonstrated remarkable reductions of Lp(a) levels of 80% (Pelacarsen) (40), 101.1% (Olpasiran) (41) and 94% (Lepodisiran) (42). Ongoing phase III clinical trials [Lp(a)HORIZON (39), OCEAN(a) Outcomes (https://clinicaltrials.gov/study/NCT05581303) and ACCLAIM-Lp(a) (https://clinicaltrials.gov/study/NCT06292013)] will assess the efficacy of the therapies in reducing Lp(a) levels.

Despite current development in Lp(a) lowering therapies, it is not clear how much of a reduction in Lp(a) is needed for a clinically relevant reduction in cardiovascular events (9). The results of ongoing phase III trials will be critical in determining whether these reductions translate into meaningful clinical outcomes, such as fewer MACE events. As the evidence base grows, these therapies may soon offer targeted treatment options for individuals with elevated Lp(a).

Importantly, the establishment of widespread Lp(a) testing is an essential first step in identifying which individuals will require Lp(a) lowering treatments. Consistency between Lp(a) measurements will prove fundamental to tracking reductions in Lp(a) and will be critical in proving the efficacy of Lp(a) lowering drugs. Pharmaceutical companies should be aware of the issues regarding Lp(a) measurement and should ensure they use the most appropriate Lp(a) assays when assessing treatment efficacy.

## Conclusion

**Figure 2:**
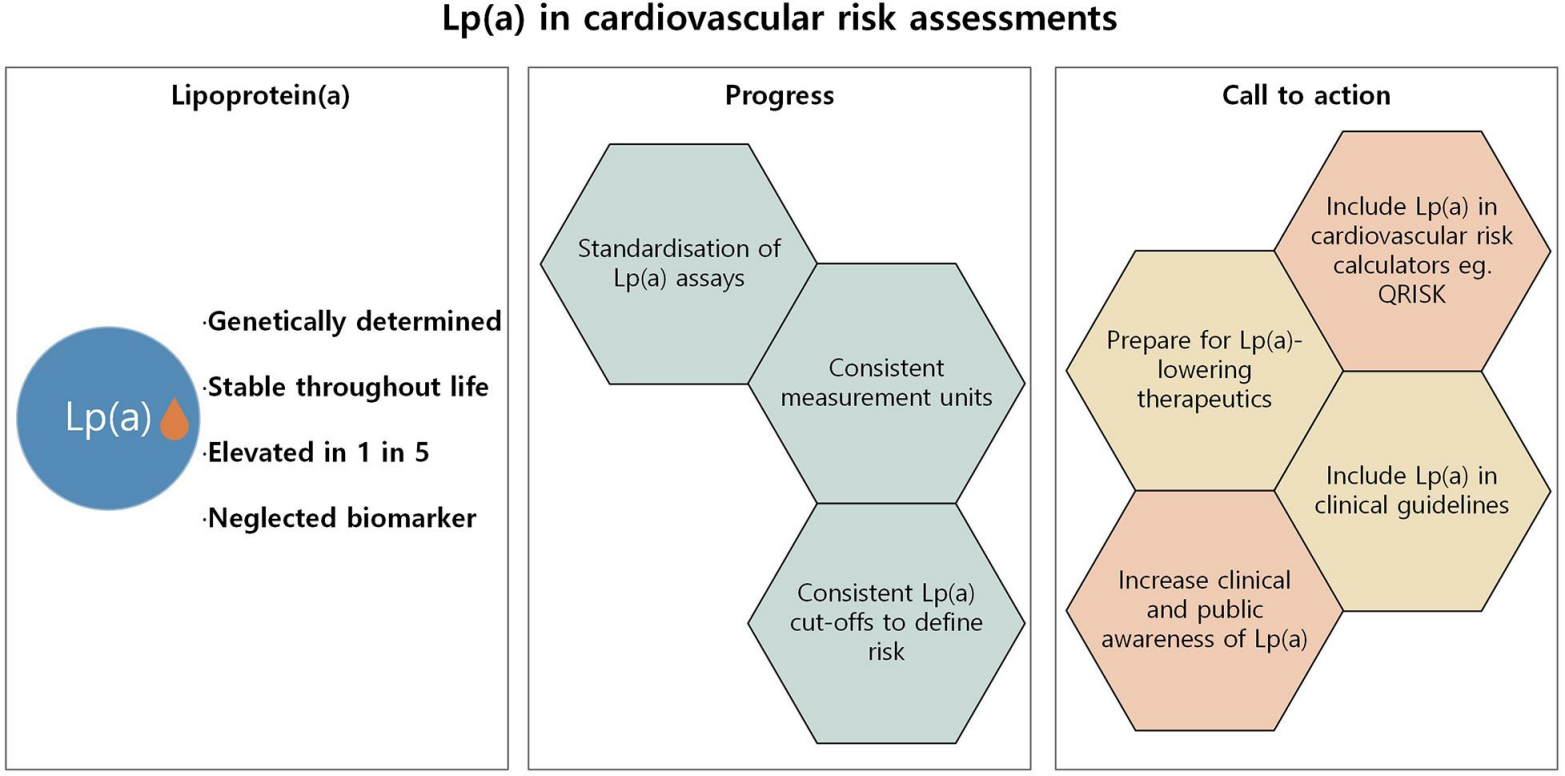
Summary of progress and further action required to integrate Lp(a) into cardiovascular risk assessments.

Lp(a) represents a critical, yet often overlooked, component of cardiovascular risk (Figure 2). As a genetically determined and largely unmodifiable risk factor, elevated Lp(a) poses a significant threat to heart and vascular health, independent of traditional lipid markers. The Brussels International Declaration has made the call clear: integrate Lp(a) into global cardiovascular health strategies, establish testing policies, and ensure every individual is tested at least once. Cardiovascular risk assessments must evolve to include Lp(a) allowing for proactive intervention which is key to preventing life-altering cardiovascular events for millions of individuals worldwide. There is a need now to increase awareness among clinicians and the general public of Lp(a) as an essential marker of cardiovascular health. With improved awareness, evolving clinical guidelines, and promising therapies on the horizon, now is the time for Lp(a) to become an integral part of cardiovascular risk assessments.

## Data Availability

The raw data supporting the conclusions of this article will be made available by the authors, without undue reservation.
