# Supplementary material for: Lipoprotein(a): the neglected risk factor in cardiovascular health
Source: Front Cardiovasc Med. 2026 Jan 9;12:1710557. doi: 10.3389/fcvm.2025.1710557 (PMC12827608; doi:10.3389/fcvm.2025.1710557)
Supplement: Supplementary file 1 [file Table1.docx]

**Supplementary Table 1:**

**Current and Emerging Lp(a)-Lowering Therapies**

| **Drug** | **Type** | **Mechanism** | **Trial Phase** | **Manufacturer** |
| --- | --- | --- | --- | --- |
| Pelacarsen | Antisense oligonucleotide | Silences LPA gene | Phase 3 (Lp(a)HORIZON)^1^ | Novartis/Ionis |
| Olpasiran | siRNA | Silences LPA gene | Phase 3 (OCEAN(a))^2^ | Amgen |
| Lepodisiran | siRNA | Silences LPA gene | Phase 3 (ACCLAIM-Lp(a)^3^ | Eli Lilly and Company |
| Muvalaplin^4^ | Oral small molecule | Blocks apo(a)-apoB100 interaction | Phase 3 (MOVE-Lp(a))^4^ | Eli Lilly and Company |
| Zerlasiran^5^ | siRNA | Silences LPA gene | Phase 2 (ALPACAR)^5^ | Silence Therapeutics |

^1^Lp(a)HORIZON; ClinicalTrials.gov ID: NCT04023552; Assessing the Impact of Lipoprotein (a) Lowering With Pelacarsen (TQJ230) on Major Cardiovascular Events in Patients With CVD (Lp(a)HORIZON).

^2^OCEAN(a); ClinicalTrials.gov ID: NCT05581303; Olpasiran Trials of Cardiovascular Events and Lipoprotein(a) Reduction (OCEAN(a)) - Outcomes Trial.

^3^ACCLAIM-Lp(a); ClinicalTrials.gov ID: NCT06292013; A Study to Investigate the Effect of Lepodisiran on the Reduction of Major Adverse Cardiovascular Events in Adults With Elevated Lipoprotein(a) - ACCLAIM-Lp(a).

^4^MOVE-Lp(a); ClinicalTrials.gov ID: NCT07157774; Assessing the Impact of Muvalaplin on Major Cardiovascular Events in Adults With Elevated Lipoprotein(a) (MOVE-Lp(a)).

^5^ALPACAR; ClinicalTrials.gov ID: NCT05537571; Evaluate SLN360 in Participants With Elevated Lipoprotein(a) at High Risk of Atherosclerotic Cardiovascular Disease Events.
